# Editorial: Nutrition, bone health, and cardiometabolic risk in childhood

**DOI:** 10.3389/fnut.2023.1229753

**Published:** 2023-06-19

**Authors:** Mariana De Santis Filgueiras, Mariane Alves Silva, Lara Gomes Suhett

**Affiliations:** ^1^Department of Nutrition and Health, Universidade Federal de Viçosa, Viçosa, Brazil; ^2^Nutrition Faculty, Universidade Federal de Mato Grosso, Cuiabá, Brazil; ^3^Department of Nutrition Sciences, Drexel University, Philadelphia, PA, United States

**Keywords:** diet, child, adolescent, bone, obesity, vitamin D

## 1. Introduction

**Figure 1:**
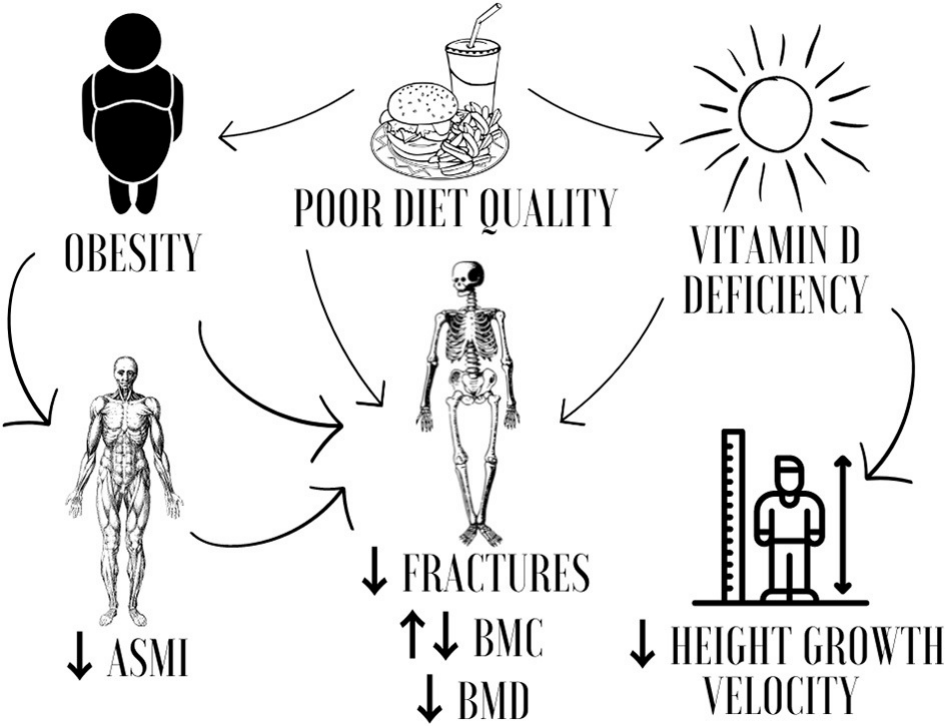
Nutrition, bone health, and cardiometabolic risk in childhood. ASMI, appendicular skeletal muscle mass index; BMC, bone mineral content; BMD, bone mineral density.

The prevalence of overweight and obesity in young people is rapidly increasing worldwide. It is estimated that there will be about 206 million children and adolescents aged 5–19 years with obesity in 2025 and 254 million in 2030 (1). This increase has been associated with physical inactivity and poorer diet quality (2) which may also contribute to adverse bone outcomes in pediatric people (3). In this context, childhood obesity has been described as a cardiometabolic risk factor of great magnitude in public health, with negative implications for children's bone health (4). Figure 1 summarizes the relationships between nutrition, bone health, and cardiometabolic risk in children and adolescents.

Frontiers in Nutrition published four articles that evaluated the association between bone mineral content (BMC) and obesity (*n* = 2); vitamin D supplementation and overweight (*n* = 1); and vitamin D levels, height growth velocity, and bone mineral density (BMD) (*n* = 1) in children and adolescents. The studies were randomized controlled trials (*n* = 2), cohorts (*n* = 1), and cross-sectional design (*n* = 1).

## 2. Obesity and bone mineral content

The relationship between overweight/obesity and bone health in children and adolescents may be attributed to change in BMC. Studies have shown that obese children had higher BMC compared to non-obese children [(5); Liang et al.]. On the other hand, Cristi-Montero et al. identified the mediating role of the appendicular skeletal muscle mass index (ASMI) in the inverse association between body fat and BMC in Chilean adolescents. This mediation was slightly higher in girls and adolescents with normal weight. Muscle tissue appears to play a role in preventing excess body fat and impaired bone health (6).

As well as for the BMC, evidence on the occurrence of fractures is contradictory. Liang et al. showed that childhood obesity was associated with a reduced risk of fractures in adulthood, while other studies indicated that overweight/obesity was associated with a higher prevalence of fractures, suggesting worse bone quality in children (7, 8). This may

be a result of the greater mechanical load on the bone structure of people with obesity (9) and the imbalance in the secretion of leptin and adiponectin by bone marrow adipocytes, contributing to an increase in osteoblasts (10).

## 3. Vitamin D supplementation and overweight

In addition to the changes in the BMC, the relationship between overweight/obesity and bone health may be attributed to vitamin D deficiency, since its prevalence is higher in people with obesity (11). However, overweight may influence the response to vitamin D supplementation in people with a genetic predisposition to lower serum levels of the vitamin D (12, 13). Asghari et al. investigated the influence of the rs2282679 polymorphism on the response of overweight and obese schoolchildren to vitamin D supplementation and identified that there was no interaction between genotype, supplementation, and vitamin D levels after the intervention. The researchers believe that obesity may nullify the association between genetic predisposition and response to vitamin D supplementation.

## 4. Vitamin D levels, height growth velocity, and bone mineral density

The vitamin D deficiency is linked to pediatric rickets, which may be directly related to child height growth. In this sense, Xiao et al., when conducting a prospective cohort of 10,450 Chinese children, displayed a non-linear and inverse L-shaped association between serum 25-hydroxy vitamin D [25(OH)D)] concentration and height growth velocity, leveling off to 40–60 nmol/L. Although some previous studies did not show an association between vitamin D and height growth (14, 15), we emphasize that the study by Xiao et al. is prospective, population-based, and with a large sample size. In addition, the investigations have divergences in the form of assessment of height growth, classification of vitamin D levels, and adjustment variables in statistical analyses.

Additionally, Xiao et al. showed that vitamin D sufficiency was associated with a reduced risk of low BMD in children; however, this result was not found among those with overweight and obesity. The authors attributed this difference according to weight status to decreased bioavailability of vitamin D in people with excess body fat, and to increased metabolic clearance of vitamin D due to increased absorption by adipose tissue.

## 5. Diet, bone health, and cardiometabolic risk

Even though the articles included in this topic did not evaluate the relationship between diet and bone health in pediatric people, it is important to holistically understand the role of diet quality on bone health using different approaches such as dietary patterns, scores, and indices (3). A recent systematic review gathered the available evidence on the association between diet quality and bone health markers in young people. The authors concluded that healthy dietary patterns exert synergistic positive effects on markers of bone health (BMC and BMD), while poor diet quality negatively impacted the bone health in children and adolescents. Additionally, they highlighted that longitudinal research using a specific tool to assess diet quality in relation to bone health is required and further studies should include bone-regulating hormones and markers of bone turnover in the analysis (3).

Diet is an important modifiable risk factor for chronic diseases, such as obesity (16), and childhood is a crucial phase for the formation of healthy eating habits (17), as well as for the adequate bone mineralization (18). Therefore, investigate the role of diet quality on bone health in the beginning of life can guide actions to prevent bone diseases and their comorbidities in the future.

## Author contributions

All authors listed have made a substantial, direct, and intellectual contribution to the work and approved it for publication.
